# Localized chronic iron deposition within non-reperfused myocardial infarctions

**DOI:** 10.1186/1532-429X-17-S1-O13

**Published:** 2015-02-03

**Authors:** Avinash Kali, Ivan Cokic, Hsin-Jung Yang, Richard Tang, Rohan Dharmakumar

**Affiliations:** 1 Cedars-Sinai Medical Center, Los Angeles, CA, USA

## Background

Chronic iron deposition is known to occur within reperfused hemorrhagic myocardial infarctions (MIs), and could be a source of prolonged inflammatory activity within MI territories. However, whether the stagnant blood downstream to the permanent occlusion (i.e. in territories of non-reperfused MI) could lead to chronic iron deposition is unknown. In this study, using a controlled canine model, we investigated whether chronic iron deposition could occur in non-reperfused MIs using T2* CMR and validated our findings with standard histological methods.

## Methods

Canines (n=19) underwent permanent ligation of the LAD and were recovered for 7 days. Breath-held, ECG-gated, 2D short axis T2* and late-gadolinium (LGE) images were acquired as contiguous slices on a clinical 3T system (Verio, Siemens Healthcare, Erlangen, Germany) at 7 days (acute) and 4 months (chronic) post-MI. T2*-weighted (multi-gradient echo; TR=12ms; 6 TEs ranging from 2.4ms to 9.9ms with ΔTE=1.5ms; flip angle=10°) and LGE images (IR-prepared FLASH; TI optimized to null remote myocardium; TR/TE=3.5/1.75ms; flip angle=25°) were acquired. Commonly used imaging parameters were slice thickness = 6mm and in-plane resolution = 1.3x1.3mm^2^. Presence of iron within MI territories was detected on T2*-weighted images using the Mean - 2 Standard Deviations criterion relative to a reference ROI drawn in the remote myocardium. Subsequently, animals were euthanized and myocardial slices were stained with TTC to visualize gross histological evidence of chronic iron deposition. Perl's staining was performed for histopathological validation of iron deposition.

## Results

Significant T2* losses were observed within the MI territories in 13 of the 19 canines at 7 days post-MI, and in all canines at 4 months post-MI. At day 7 post-MI, mean T2* value of the MI was 42.6±10.5% lower than that of remote myocardium (16.7±3.4ms vs. 28.8±3.0ms, p<0.01); and at month 4 post-MI, T2* value of the MI was 39.1±12.1% lower than that of remote myocardium (19.3±4.1ms vs. 31.7±1.8ms, p<0.01). Mean MI and iron volumes (as percentage of total LV volume) were as follows: day 7 post MI - 14.7±8.7% and 2.1±1.6% respectively; 4 months post MI - 7.2±5.6% and 1.9±1.1%. Ex-vivo TTC staining at 4 months post-MI showed pale brown discoloration within the infarct core indicating chronic iron deposition. Perl's stain confirmed CMR findings of iron deposition within chronic MI territories.

## Conclusions

These studies demonstrated for the first time that (a) T2* signal losses within acute MI territories are not limited to hemorrhagic infarctions; and (b) chronic iron deposition can occur within non-reperfused MIs. The long-term effects of these depositions in the infarcted heart remain to be investigated.

## Funding

This work was supported in part by grants from American Heart Association (13PRE17210049) and National Heart, Lung, And Blood Institute (HL091989).

**Figure 1:**
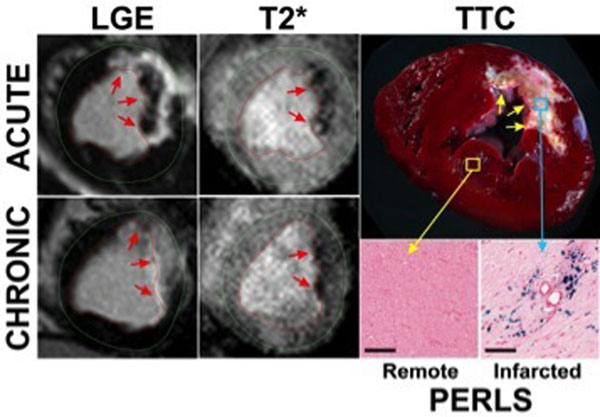
Representative LGE images and T2*-weighted images (TE=6.9ms) acquired on day 7 (acute) and at 4 months (chronic) post-MI from a canine subjected to permanent ligation of LAD are shown. Arrows point to the site of infarction on LGE images and iron deposition on T2*-weighted images. T2* losses were observed within infarcted territories in both acute and chronic phases of MI indicating iron deposition. Ex-vivo TTC staining showed pale brown discoloration in the core of MI. Microscopic histopathology using Perls staining (scale bars = 200µm) showed significant iron deposition within infarcted territories but not in remote myocardium.

